# Supplementary material for: Nitrogen fertilizer amount has minimal effect on rhizosphere bacterial diversity during different growth stages of peanut
Source: PeerJ. 2022 Nov 2;10:e13962. doi: 10.7717/peerj.13962 (PMC9636873; doi:10.7717/peerj.13962)

**Supplementary Materials**

**Nitrogen fertilizer amount has minimal effect on rhizosphere bacteria diversity during different growth stages of peanut**

Zheng Yang^1^, Lin Li ^2^, Wenjuan Zhu ^1^, Suyuan Xiao ^1^, Siyu Chen ^1^, Jing Liu ^1^, Qian Xu ^1^, Feng Guo ^3*^, Shile Lan ^1*^

^1^ College of Bioscience and Biotechnology, Hunan Agricultural University, Changsha 410128，China

^2^ College of Agronomy, Hunan Agricultural University, Changsha 410128, China

^3^ Biotechnology Research Center, Shandong Academy of Agricultural Sciences/Shandong Key Laboratory of Crop Genetic Improvement and Ecological Physiology, Jinan 250100, China

Corresponding author: S. Lan, e-mail:875540378@qq.com; F. Guo, e-mail: guofeng08-08@163.com.

Figure S1. Pearson correlation of soil chemical properties. The red numbers in the upper-right indicate positive correlation and the blue numbers indicate negative correlation. The lower-left shows the scatter charts of the data. AP, available phosphorus; TP, total phosphorus content; AK, available potassium; TK, total potassium content; TN, total nitrogen content; ECa, exchangeable calcium; AN, alkali-hydrolyzable nitrogen. * p < 0.05; **, p < 0.01; ***, p < 0.001.


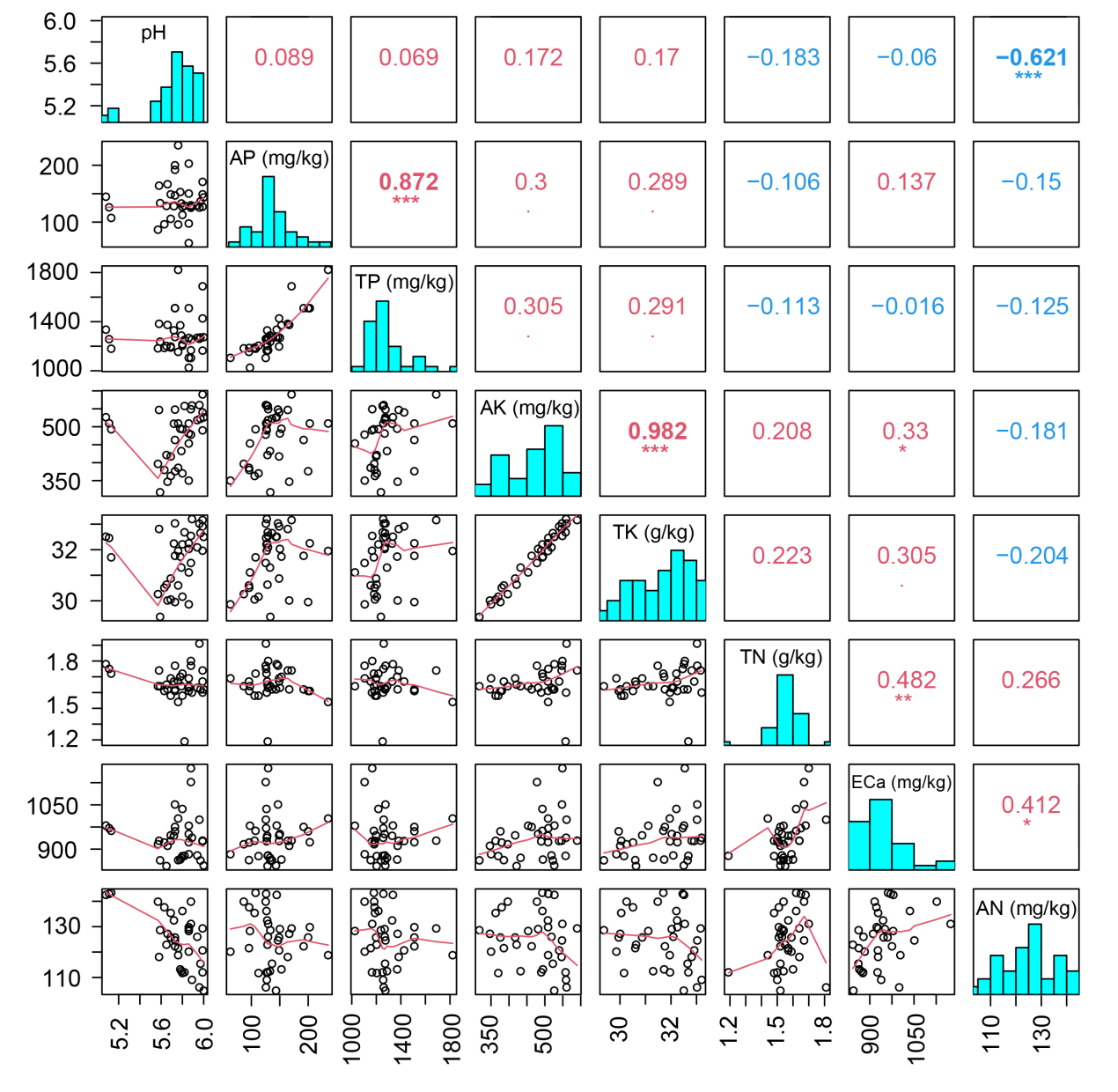


Figure S2. LEfSe results showed significantly different genera in peanut rhizosphere microbiota among different amounts of N-f fertilizer application of all samples (A and B), seedling stage samples (C and D), flower needle stage (E and F), and mature stage (G and H). UB, uncultured bacterium.


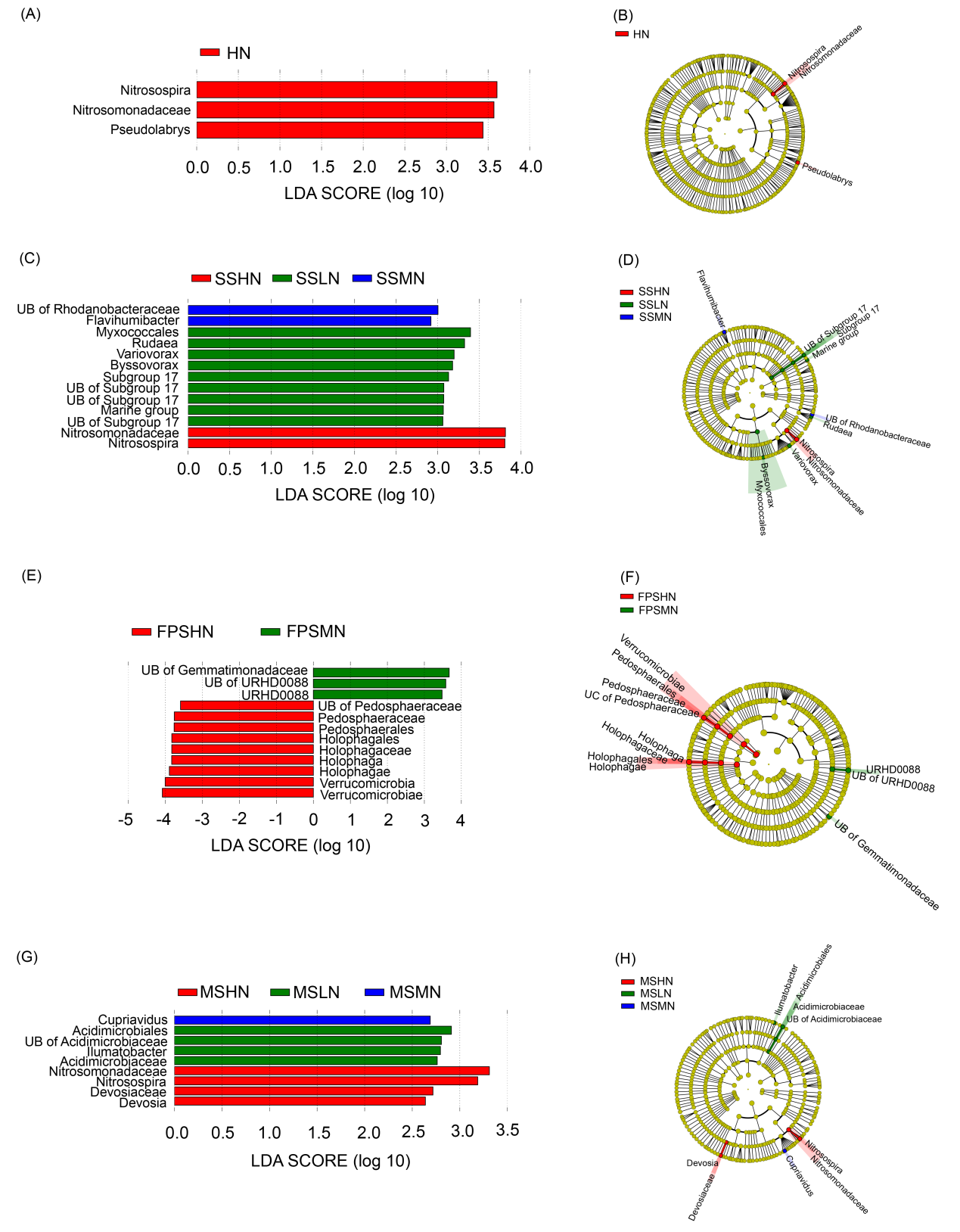

Supplement: Supplemental Information 1 [file peerj-10-13962-s001.docx]
